# Prevalence of the Metabolic Syndrome in Central and South American Immigrant Residents of the Washington, DC, Area

**DOI:** 10.1155/2017/9531964

**Published:** 2017-07-04

**Authors:** Regina M. Gill, Saira A. Khan, Robert T. Jackson, Marguerite Duane

**Affiliations:** ^1^Department of Nutrition and Food Science, University of Maryland, College Park, MD 20742, USA; ^2^Department of Family Science, Georgetown University, Washington, DC 20007, USA

## Abstract

The objective of this study was to estimate the prevalence of Metabolic Syndrome (MetS) and its risk components and then compare differences in the risk components among low-income, uninsured Central and South American recent immigrants to the USA. This cross-sectional survey sampled 1,042 adult patients from a medical clinic in metropolitan Washington, DC. The overall prevalence of the MetS was 26.9% estimated using the modified harmonized definition. The most common abnormal metabolic indicator for women was an elevated BMI ≥ 30 kg/m^2^ (36.1%), while, for men, it was an elevated triglyceride level (46.5%). The risk of abnormal MetS indicators increased steadily with increasing BMI. The abnormal indicator combination identifying the most subjects with the MetS included the following: high triglycerides, low HDL cholesterol, and obesity. MetS rates were highest among subjects from El Salvador and Honduras, 31.3% and 28.0%, respectively, and lowest among subjects from Bolivia (21.7%). Dyslipidemia and high BMI increased the likelihood of having the MetS, which is consistent with studies on Mexican Americans in the San Antonio Heart Study and studies within Central and South American countries. This study adds new baseline epidemiological data for largely understudied, low-income, and mostly recent immigrant groups.

## 1. Introduction

Cardiovascular diseases (CVD) are the leading causes of death among Americans, including one of the fastest growing segments of the United States of America (USA) population, namely, Hispanics [1]. Central Americans are the fastest growing group among Hispanics in the USA and are largely understudied.

Metabolic Syndrome (MetS) is a cluster of metabolic abnormalities that each has been associated with an increased risk of developing CVD and Type 2 Diabetes Mellitus (T2DM) [2–6]. The characteristics of the syndrome include glucose intolerance, hyperinsulinemia, high plasma triglycerides, decreased high density lipoprotein (HDL) cholesterol, hypertension, and central obesity [7]. Having any three of the five abnormal indicators above has been defined as MetS [8]. It has been closely associated with a generalized metabolic disorder called insulin resistance, in which tissue responsiveness to the normal action of insulin is impaired [5]. Although the cause of MetS has not been definitively established, overweight/obesity, physical inactivity, certain dietary patterns [9, 10], and genetic factors are considered major risk factors [6, 9, 10].

Much of what we know about the epidemiology of chronic diseases in the USA comes from large national studies such as the National Health and Nutrition Examination Survey (NHANES), which is conducted to ascertain the health and nutrition status of non-Hispanic whites, non-Hispanic blacks, and Hispanic Americans of Mexican descent, living in the USA. Central and South American groups that more recently arrived to the USA have not been studied. Based on the findings from the NHANES, the prevalence of MetS among the US adult population was 34.0% (men [35%] and women [33%]). Mexican Americans in the US reported a similar prevalence (33%). Despite the plethora of information on the Hispanic groups studied by NHANES, generalizations cannot be made to other Hispanic subgroups. According to Nath (2005) [11], “findings from one Hispanic subgroup cannot be applicable or extrapolative to other Hispanic subgroups because each subgroup's social histories, cultural identities, health behaviors, and genetic compositions are unique.” The objective of this study was to estimate the percent of MetS in Central and South Americans by gender and age and to rank the risk components from most common to least common. Additionally, we examined the relationship between MetS and various demographic variables.

## 2. Methods

A cross-sectional survey was conducted among subjects attending a Catholic medical clinic in Washington, DC. This clinic serves Hispanics and other immigrant communities. Data were obtained from files of the medical clinic, which is located in a predominantly Hispanic neighborhood. Clinic services are usually free or of low cost. The final sample consisted of 1,042 adults aged ≥ 18 years, who reported being from any Central or South American country and who had fasting lipid profile measurements available.

Data were abstracted onto structured questionnaires using systematic sampling, where the first subject was chosen at random and each additional 3rd subject was subsequently sampled until 1042 subjects were obtained. The calculated sample size was 1014 subjects based on 3% margin of error, 95% confidence, and 50% expected MetS prevalence. Protocols were approved by the medical Catholic Charities Review Board and by the Institutional Review Board at the University of Maryland.

Sociodemographic variables included age, gender, country of origin, state of residence, education, employment status, and years of residence in the USA. Medical history was used to identify subjects diagnosed by a physician with diabetes, hypertension, or heart problems.

Biochemical indices were evaluated by a commercial laboratory. Blood glucose was analyzed using an automated enzymatic method [12]. Cholesterol and HDL cholesterol were both analyzed enzymatically. Triglyceride (TG) levels were analyzed by automated spectrophotometry [13]. Low density lipoprotein-cholesterol was mathematically derived using the Friedewald formula for subjects with TG levels ≤ 400 mg/dL [14].

The MetS was defined using a modified harmonized definition that identified subjects with three or more of the following: (1) Body Mass Index (BMI) ≥ 30 kg/m^2^, (2) fasting plasma glucose ≥ 100 mg/dl, (3) HDL cholesterol < 40 mg/dl for men and < 50 mg/dl for women, (4) TG ≥ 150 mg/dl, and (5) blood pressure: systolic blood pressure (SBP) ≥ 130 or diastolic blood pressure (DBP) ≥ 85 mmHg [15]. Waist Circumference (WC) is not routinely collected in the medical clinic, so we used weights and heights to calculate BMI. In this study, we used the WHO BMI cutoff [16]. BMI was calculated as weight in kg/height in meters^2^ and was categorized as follows: underweight (<18.5 kg/m^2^), normal weight (18.5–24.9 kg/m^2^), overweight (25.0–29.9 kg/m^2^), and obese (≥30.0 kg/m^2^), according to WHO criteria [17]. Underweight subjects (BMI < 18.5 kg/m^2^) and subjects receiving insulin therapy for treatment of diabetes were excluded.

### 2.1. Statistical Analysis

Data are presented as means ± standard deviations (SD) for continuous variables and as frequencies and percentages for categorical variables. Differences between mean values of MetS components based on country of origin and BMI category were assessed by analysis of variance (ANOVA), with a Tukey-Kramer post hoc test to accommodate groups with unequal sample sizes. Analysis of covariance (ANCOVA) was also used to control for age when means of lipid values were compared among groups. Chi Square test was used to compare prevalence rates of MetS and MetS components in analyses stratified by gender and BMI category. Student's* t*-test compared independent means between men and women for selected variables. Partial correlation was used (controlling for age) when studying bivariate relationships. All tests were two-tailed. Significance level was set at *P* ≤ 0.05; analyses were performed using Statistical Analysis System (SAS) (Version 9.4; SAS Institute Inc., Cary, NC).

## 3. Results

A total of 1,042 males (*n* = 338) and females (*n* = 704) were chosen for our study. The mean age of the sample was 42 ± 13 years and ranged between 18 and 87 years. Nearly 50% of the total sample were from El Salvador, 57% were Maryland residents and had a median income of $13,000 per year, and English was spoken by 23%. Forty-five percent of subjects were married and completed primary and secondary education (69%). Only 11% (*n* = 100) of the sample had medical insurance. The mean number of years that subjects had lived in the USA was 8.8 years.

Table 1 shows the anthropometric, biochemical, and clinical characteristics of the subjects stratified by gender. Females had a significantly higher BMI than males (*P* ≤ 0.05). The mean TG value and LDL cholesterol in males exceeded the harmonized cutoff levels (Table 1). LDL cholesterol for females also exceeded cutoff levels. 78.4% of males and 74.9% of females were overweight and 28.4% of males and 36.1% of females were obese.

The overall prevalence of MetS among the five major Hispanic groups in our study was 28.9%.  Table 2 shows the overall prevalence of MetS among subjects from the five most frequently reported countries represented in this study (El Salvador, Honduras, Peru, Guatemala, and Bolivia). The overall percent of MetS was similar among subjects from El Salvador and Honduras, with rates of 31.3 and 28.0%, respectively. Bolivian subjects had a somewhat lower MetS percent of 21.7%.

Table 3 shows the percent of individual MetS components among males and females. The most common abnormal lipid indicator of MetS was high triglyceride level and low HDL cholesterol for males and females, respectively. More females (50.7%) had low levels of HDL cholesterol than males (43.2%). The overall percentage of hypertriglyceridemia was 37%.

The combined percent of hypertension was 24.9%. About 14% (13.96%) of men had elevated SBP and 12.5% had elevated DBP. The percentages of females with elevated SBP and DBP were 12.9% and 9.7%, respectively. Overall, 28.3% of the males and 23.3% of the females were hypertensive.

Among males, 20.7% had one abnormal metabolic component, 26.0% had two abnormal components, and 18.3%, 5.3%, and 1.2% had three, four, or all five abnormal components of the MetS. Females had lower percentages of abnormal MetS indicators, with values of 29.7, 19.9, 14.8, 4.8, and 0.4%, respectively.

Table 4 shows MetS risk components by BMI category. Across all MetS components (TG, HDL, BP, and blood glucose), obese subjects (BMI of  ≥30 kg/m^2^) had significantly higher percentage rates than did nonobese subjects (BMI values < 30 kg/m^2^). The numbers of obese subjects meeting the harmonized criteria for each component were more than three times greater than that of subjects with normal BMI (*P* ≤ 0.05).

All possible combinations of three of the five modified harmonized criteria used to diagnose MetS are shown in Table 5. The presence of MetS varied significantly by the specific criteria used. Obesity (BMI ≥ 30 kg/m^2^), in combination with hypertriglyceridemia and low HDL cholesterol, was the most prevalent contributor to the MetS for both genders.

There were no significant associations for MetS by age, sex, years in USA, education, income, and ethnic group.

## 4. Discussion

CVD are the leading causes of death in the USA. Low-income individuals, especially those who lack health insurance, are at greater risk for morbidity and mortality from chronic diseases [18]. MetS presents a major risk for the development of CVD and T2DM. There is a paucity of data on the MetS and its components among the growing numbers of low-income Central and South Americans, particularly for recent immigrants to the USA.

Our study provides one of the first comprehensive pieces of data on these hard-to-reach and underserved groups and showed that only 11% of these subjects have health insurance. The overall prevalence of the MetS in this sample was 26.9%. The prevalence in men was 31.0%, while the prevalence in women was 25.0%. Lorenzo et al. (2007) [19], using data from the 1999–2002 NHANES, examined the prevalence of the MetS (using NCEP ATP III criteria) in Mexican American adults and found that 32.2% and 37.8% of Mexican American men and women, respectively, had the MetS [19]. In the San Antonio Heart Study (SAHS) on the other hand, Lorenzo et al. (2006) [20], also using NCEP criteria, found the prevalence of the MetS to be 29.6% for men and 30.9% for women among Mexican Americans. Our study may have a lower percent of MetS in women because we had data on adults from 18 to 87 years while the San Antonio Heart Study had ages of 25–64 years.

In a study of US adults aged ≥ 20 years, the prevalence of hypertriglyceridemia, low HDL cholesterol, hypertension, and high blood glucose were 40.2%, 34.1%, 39.8%, and 21.1%, respectively, among Mexican American men and 35.8, 46.6, 32.9, and 32.9%, among Mexican American women, respectively [4]. The subjects of our study had a slightly lower overall prevalence of the MetS; however, unlike the studies based on the NHANES and SAHS data, the prevalence of MetS in our study was lower in women than in men. The NHANES data are based on representative samples of the population while our data are from a convenience sample of individuals attending clinics where obesity may be more prevalent and our subjects are older.

In our sample, the most prevalent metabolic abnormalities were elevated BMI (obesity) (28.4% men and 36.1% women), hypertriglyceridemia (46.5% men and 32.5% women), and low HDL cholesterol (43.2% men and 50.7% women). The percent of subjects with high FPG was low despite the large percentage (78%) of overweight subjects. Previous studies have shown that heavier individuals are more likely to be insulin-resistant and that adiposity and insulin resistance are highly correlated [10, 20–22]. Vazquez et al. (2007) [21] found that general obesity is a good predictor of diabetes among white US and European populations. Our results showed that in general the higher the level of adiposity, the higher the prevalence of each MetS risk component (Table 3). Obese subjects had significantly higher MetS rates than overweight or normal weight subjects. Han et al. (2002) [2] found in an analysis of Mexican American SAHS subjects that the odds of developing metabolic disorders, such as hypertriglyceridemia, low HDL cholesterol, hypertension, and Type 2 DM, increased with a BMI ≥ 30 kg/m^2^ for men and women. However, they also found the odds of having low HDL cholesterol were greater for overweight as opposed to obese women, which differs from our findings.

In a population-based study in Peru, PREVENCION, the most common metabolic abnormality of women was low HDL cholesterol (60.9%), whereas, in men, it was hypertriglyceridemia (52.0%) followed by low HDL cholesterol (32.5%). The abnormalities found in the PREVENCION study are similar to those found in our study, including large numbers of men and women with high BMI values. Like us, they also found that abnormal FPG was the least common MetS component for men (5.4%) and women (5.0%) [23].

Espinosa-Larrañaga et al. (2005) [22] estimated that approximately 50% of the populations in Argentina, Chile, Paraguay, Peru, and Colombia are overweight and more than 15% are obese. They also found that, in Chile, 39.3% of the population had low HDL cholesterol and, in Venezuela, men had higher levels of triglycerides (47%) with lower levels of HDL cholesterol (40%), which are both consistent with our findings.

In our study, dyslipidemia (elevated triglycerides and decreased HDL cholesterol levels) was common. Similar patterns of dyslipidemia have been found in Venezuelan [24] and Costa Rican [9] subjects. Martínez-Ortiz et al. (2006) [9] implicate Latin American dietary patterns as a possible contributor to the presence of dyslipidemia and found a positive association between diet and dyslipidemia. They indicate that a “staple” dietary pattern, characterized by an increased intake of refined grains (white bread and rice), added sugar, coffee, legumes, red meat, and increased use of palm oil for cooking, was associated with lower HDL cholesterol and increased risk of myocardial infarction in Costa Rican adults [9]. Some of the groups we studied share a similar diet pattern to that detailed by Martínez-Ortiz et al. (2006) [9]; however we did not study diet and therefore cannot link the observed similar pattern of dyslipidemia with any one diet type.

Obesity, low HDL cholesterol, and high triglyceride levels were the indicators that identified the most subjects with the MetS (Table 4). Combinations using elevated blood pressure identified fewer subjects with the MetS.

Hypertriglyceridemia, low HDL cholesterol, and hypertension are commonly found in those who are insulin-resistant [22]. However, in Central Americans this combination of criteria did not identify as many subjects as the criteria that included BMI. Overall, we found that 28.3% of the men and 23.3% of the women were hypertensive, results which are similar to those found by others in adult Latin American populations [23, 24]. The combinations which include dyslipidemia and obesity are especially detrimental because they are associated with Type 2 DM and an increased risk of cardiovascular events [22].

Five countries made up the largest numbers of subjects in our study. Subjects from El Salvador (*n* = 508) had the highest percent of MetS (31.3%), while those from Bolivia (*n* = 60) had the lowest percent (21.7%). Our findings suggest that Bolivians have lower risks associated with the MetS. However, this difference may be due to the small sample size of Bolivian subjects.

Strengths of this study include its focus on and addition of data on an understudied, but rapidly increasing, segment of the US Hispanic population. The clinic that we chose for this study serves a predominantly large Central and South American population and they were our target subjects. The use of systematic sampling allowed for data to be representative of the clinic patients. Limitation of this study was that waist circumference was not collected as a MetS risk component. However, other organizations such as the World Health Organization have also used BMI, instead of WC, to define MetS. Although the clinics readily collect the data needed for calculating MetS, another limitation was that we drew our sample from the clinic rather than the community which the clinics support.

This study provides new data on understudied groups of Hispanics and shows that there are disparities in MetS risk among these Hispanic subgroups and between these groups and the more established Hispanic groups that are long-term citizens and residents of the USA. Recent immigrants and low-income individuals without health insurance (89% of our sample) are especially vulnerable. Lack of insurance adversely impacts access to timely preventive and curative services needed to effectively control this emerging health crisis in these communities. Clinicians and public health authorities need to be aware of the differing percent of MetS in the different immigrant subgroups and what the most likely clustering of adverse indicators are among these subgroups.

These results suggest that for both prevention and intervention knowledge of the clustering of the metabolic indicators is important. The clustering profile differed substantially across the ethnic group. Knowledge of clustering is important for clinicians to identify the interventions that are most distinctive for the specific ethnic group. Knowledge of the clustering could assist in management of metabolic indicators. Clinicians need to take into account the ethnic group of the Hispanics before intervention efforts.

## Figures and Tables

**Table 1:** Background characteristics of the Central and South American subjects by gender^1^.

	*n*	Male	*n*	Female
Age (years)	338	41.5 ± 13.6	704	42.8 ± 13.2
≤34 (%)	114	34^2^	237	34^2^
35–44 (%)	96	28^2^	166	24^2^
45–54 (%)	66	20^2^	173	25^2^
55–64 (%)	46	14^2^	90	13^2^
≥65 (%)	16	5^2^	38	5^2^
Weight (kg)	331	77.3 ± 12.5	701	69.3 ± 14.2
Height (cm)	323	165.4 ± 7.4	688	154.7 ± 6.9
BMI (kg/m^2^)	323	28.3 ± 4.1	688	29.0 ± 5.6
SBP (mmHg)	336	119.7 ± 17.7	703	115.7 ± 18.5^3^
DBP (mmHg)	336	72.9 ± 12.1	703	71.0 ± 10.9^3^
TC (mg/dL)	332	191.5 ± 38.9	694	190.5 ± 36.7
HDL-C (mg/dL)	332	42.9 ± 10.4	694	50.5 ± 12.7^3^
LDL-C (mg/dL)	320	116.7 ± 33.5	684	113.3 ± 31.3
TG (mg/dL)	331	163.8 ± 97.9	691	136.4 ± 78.6^3^
FPG (mg/dL)	329	99.9 ± 31.3	686	93.3 ± 27.9^3^

^1^Data are mean ± SD, unless otherwise specified. ^2^Data are percentages (%). ^3^Significantly different from men; BMI, Body Mass Index; SBP, systolic blood pressure; DBP, diastolic blood pressure; TC, Total Cholesterol; HDL, High Density Lipoprotein; LDL, low density lipoprotein; TG, triglycerides; FPG, fasting plasma glucose.

**Table 2:** Percentage of Central and South Americans meeting Metabolic Syndrome criteria by country of origin^*∗*^.

Number of components	El Salvador	Honduras	Peru	Guatemala	Bolivia
	*n* = 508	*n* = 107	*n* = 98	*n* = 93	*n* = 60
	%	%	%	%	%
1	24.0	30.8	32.7	21.5	25.0
2	20.1	18.7	13.3	35.5	6.7
**3**	**19.9**	**19.6**	**20.4**	**16.1**	**20.0**
4	8.7	7.5	4.1	6.5	1.7
5	2.8	0.9	0	3.2	0
Prevalence	31	28	24	25	21
Total number of subjects w/MetS	159	30	24	24	13

Prevalence of MetS among the five Hispanic subgroups 28.9%. ^*∗*^Values are percentages.

**Table 3:** Prevalence of individual Metabolic Syndrome components among male and female Central and South Americans.

	Number of subjects and % with abnormal MetS components				
MetS component	Male		Female		*P*
	Number	%	Number	%	
High BMI (≥25 kg/m^2^)	265	78.4	523	74.9	0.15
High BMI (≥30 kg/m^2^)	96	28.4	254	36.1	0.01
High FPG (≥100 mg/dl)	87	25.7	112	15.9	0.04
Low HDL cholesterol^†^	146	43.2	357	50.7	<0.01
High TG (≥150 mg/dl)	157	46.5	229	32.5	<0.01
HTN (≥130/85 mmHg)	96	28.3	164	23.3	0.51
Overall prevalence of metabolic syndrome	105	31	176	25	0.28

^†^Low HDL: male < 40 mg/dl, female < 50 mg/dl; BMI, Body Mass Index; FPG, fasting plasma glucose; HDL, high density lipoprotein; TG, triglycerides; HTN, hypertension.

**Table 4:** Number of subjects and prevalence of Metabolic Syndrome components in Central and South Americans by BMI category.

MetS component	Number and percent of subjects			
	Normal	Overweight	Obese	*P* value
	18.5–24.9 kg/m^2^	25.0–29.9 kg/m^2^	≥30.0 kg/m^2^	
High FPG (100 mg/dl)	29 (13%)	70 (16%)	95 (28%)	<0.0001
Low HDL (<40 mg/dl)^†^	43 (20%)	101 (23%)	136 (39%)	<0.0001
Low HDL (<50 mg/dl)^₤^	91 (41%)	249 (57%)	248 (71%)	<0.0001
High TG (150 mg/dl)	37 (17%)	158 (36%)	173 (50%)	<0.0001
SBP (≥130 mmHg)	34 (16%)	94 (22%)	105 (30%)	0.0001
DBP (≥85 mmHg)	13 (6%)	33 (8%)	63 (18%)	<0.0001
HTN (130/85 mmHg)	34 (16%)	100 (23%)	114 (33%)	<0.0001

Data are *n* (%). ^†^Low HDL (<40 mg/dl): males. ^₤^Low HDL (<50 mg/dl): females. FPG, fasting plasma glucose; HDL, high density lipoprotein; TG, triglycerides; SBP, systolic blood pressure; DBP, diastolic blood pressure; HTN, hypertension.

**Table 5:** Identification of the Metabolic Syndrome in Central and South Americans using all combinations of any three criteria.

Criteria used^*∗*^	Number of subjects with Metabolic Syndrome									
	Male					Female				
	Normal	Overweight	Obese	*n*	*P*	Normal	Overweight	Obese	*n*	*P*
BMI, TG, HDL	0	0	42	42	<0.0001	0	0	88	88	<0.0001
TG, HDL, BP	3	16	14	33	0.20	3	18	29	50	0.0012
TG, HDL, FPG	0	10	13	23	0.006	3	11	22	36	0.0061
HDL, BP, FPG	2	5	11	18	0.006	3	7	14	24	0.014
BMI, HDL, BP	0	0	21	21	0.0001	0	0	47	47	<0.048
BMI, TG, BP	0	0	24	24	<0.0001	0	0	35	35	<0.0001
BMI, TG, FPG	0	0	24	24	<0.0001	0	0	27	27	<0.0001
BMI, FPG, BP	0	0	17	17	<0.0001	0	0	23	23	<0.0001
BMI, FPG, HDL	0	0	18	18	<0.0001	0	0	37	37	<0.0001
TG, FPG, BP	0	10	12	22	0.010	6	9	11	26	0.8510

^*∗*^BMI: men and women, ≥30.0 kg/m^2^; glucose 100 mg/dl; HDL: men < 40 mg/dl, women < 50 mg/dl; triglyceride ≥ 150 mg/dl; blood pressure ≥ 130/85 mmHg. BMI, Body Mass Index; BP, blood pressure; HDL, high density lipoprotein; TG, triglycerides; FPG, fasting plasma glucose.
